# *The Insight of In Silico* and *In Vitro* evaluation of *Beta vulgaris* phytochemicals against Alzheimer’s disease targeting acetylcholinesterase

**DOI:** 10.1371/journal.pone.0264074

**Published:** 2022-03-03

**Authors:** Sidra Rehman, Usman Ali Ashfaq, Muhammad Sufyan, Imran Shahid, Bushra Ijaz, Mureed Hussain

**Affiliations:** 1 Department of Biosciences, COMSATS University Islamabad (CUI), Islamabad, Pakistan; 2 Department of Bioinformatics and Biotechnology, Government College University Faisalabad, Faisalabad, Pakistan; 3 Faculty of Medicine, Department of Pharmacology and Toxicology, Umm Al-Qura University, Makkah, Saudi Arabia; 4 Centre of Excellence in Molecular Biology, University of the Punjab, Lahore, Pakistan; 5 Department of Life Sciences, University of Management and Technology, Lahore, Pakistan; National Cancer Institute at Frederick, UNITED STATES

## Abstract

*B*. *vulgaris* extracts possess antioxidant, anti-inflammatory along with its role in improving memory disorders. Subsequently, *in vitro* and *in silico* studies of its purified phytochemicals may expand complementary and alternative Alzheimer’s therapeutic option. Super activation of acetylcholinesterase enzyme is associated explicitly with Alzheimer’s disease (AD) ultimately resulting in senile dementia. Hence, acetylcholinesterase enzyme inhibition is employed as a promising approach for AD treatment. Many FDA approved drugs are unable to cure the disease progression completely. The Present study was devised to explore the potential bioactive phytochemicals of *B*. *vulgaris* as alternative therapeutic agents against AD by conducting *in vitro* and *in silico* studies. To achieve this, chemical structures of phytochemicals were recruited from PubChem. Further, these compounds were analyzed for their binding affinities towards acetylcholinesterase (AChE) enzyme. Pharmacophoric ligand-based models showed major characteristics like, HBA, HBD, hydrophobicity, aromaticity and positively ionizable surface morphology for receptor binding. Virtual screening identified three hit compounds including betanin, myricetin and folic acid with least binding score compared to the reference drug, donepezil (-17 kcal/mol). Further, *in vitro* studies for anti-acetylcholinesterase activity of betanin and glycine betaine were performed. Dose response analysis showed 1.271 μM and 1.203 μM 50% inhibitory concentration (IC_50_) values for betanin and glycine betaine compounds respectively. Our findings indicate that phytoconstituents of *B*. *vulgaris* can be implicated as an alternative therapeutic drug candidate for cognitive disorders like Alzheimer’s disease.

## Introduction

Neurodegenerative diseases are the diverse group of genetic disorders characterized by the loss of structure and function of neurons. Alzheimer’s disease (AD) is responsible for 60 to 80% of the total mental illness in relatively older or old aged individuals. AD is the most common form of dementia accounting for 5.5 million patients in United States alone [1]. Memory loss, personality changes, abnormal behavior and loss of thinking ability are major characteristics of AD. Early clinical symptoms include difficulty remembering names, events, conversation, short-term memory loss, mood swings and failure to perceive new information. As condition advances, late clinical symptoms become more prominent like impaired communication, poor judgement, disorientation eventually difficulty in walking, eating and swallowing [2].

Pathological data regarding AD depicts that degeneration in cholinergic neuron–rich regions is correlated with loss of memory, apathy and agitation. Acetylcholine (ACh) has significantly associated learning and memory function including memory encoding, consolidation storage and the rejuvenation process [3–5]. Multiple drug classes for AD treatment have been approved amongst the Acetylcholinesterase Inhibitors (AChEIs), the most important class of drugs. Cholinergic system depicts its major role in coordination of learning and memory key mechanisms. Several research studies have shown the role of both acetylcholinesterase (AChE) and butyrylcholinesterase (BuChE) in amyloid beta (Aβ) aggregation during early phases of amyloid plaque formation. Consequently, inhibition of AChE and BuChE tends to increase the ACh quantity in brain thereby reducing the plaque formation. BuChE, closely related to AChE, involves in ACh hydrolysis and mainly found in the peripherals including plasma therefore blocking BuChE may cause many side effects. Accordingly scientists are developing selective AChEIs to minimize these side effects [6,7]. FDA approved drugs including group of acetylcholinesterase inhibitors are being used for the treatment of AD. Donepezil, tacrine, galantamine and rivastigmine are clinically approved AChEIs with limited efficacy for AD management. Cholinergic adverse effects including insomnia, muscle cramps, nausea, hepatotoxicity, bone fracture, eczema rash, unusual weakness, gastrointestinal disturbances, nocturia are the major issues of these drugs. Therefore, development of more effective, safe and potent therapeutic agent is a need of time.

Plants utilization in traditional medicines is an important part of tradition and culture of majority of world’s population. Presence of secondary metabolites has depicted therapeutic properties of medicinal plants. Plants are the valuable sources for the development of natural therapeutic compounds. *Beta vulgaris* Linn (Chenopodiaceae) generally known as ‘beetroot’ or ‘chukandar’. It is native to Mediterranean region and expansively cultivated in Europe, America, Europe, India and Pakistan [8,9]. *B*. *vulgaris* is also known as ‘red beet’ and ‘sugar beet’ due to its color and usage in sugar industry. Based on its pharmacological and nutritive values, it is cultivated in different regions of Pakistan. Raw *B*. *vulgaris* is a rich source of folic acid and moderate source of some minerals and health beneficial secondary metabolites. Biologically valuable compounds like carotenoids [10], glycine betaine [11], betacyanins [12], flavonoids, polyphenols, betanin [13], vitamin C and folates [14] are the major components of beetroot. The presence of phenols, flavonoids and vitamin C indicates antioxidant activity of beetroots [15]. Therefore, its consumption may contribute to prevent and cure the age-related diseases. The current research study performed the virtual screening of *B*. *vulgaris* phytochemicals against AChE. Phytochemicals including betanin and glycine betaine were analyzed *in vitro* for their AChE inhibition activity and IC_50_ values were also calculated.

## Materials and methodology

### Materials

AlbuMAX™| Lipid-Rich BSA Cat#11020021, M/s Gibco™ and 5,5′-Dithiobis (2-nitrobenzoic acid) (DTNB; Ellman’s Reagent) Cat# 22582, M/s Gibco™ were purchased from Thermo Fisher Scientific. Acetylthoicholine chloride (ATCCl) Cat # A5626 M/s Sigma Aldrich, Gelatin solution Cat # G1393 M/s Sigma Aldrich, Acetylcholinesterase (AChE) from Electric eel Cat # C3389 M/s Sigma Aldrich were purchased from Sigma. Betanin Cat # B0397 was purchased from TCI America and glycine betaine Cat # B2629 was purchased from Sigma Aldrich.

### Receptor protein selection and refinement

Protein coding gene, ‘AChE’ was identified by RCSB Protein Data Base (PDB) (https://www.rcsb.org/). AChE is composed of six exons. Three AChE polypeptides are produced from alternative splicing resulting a combination of isoforms. These isoforms exhibit similar catalytic properties with different quaternary structure and distribution pattern in tissues [16–18]. AChE harbors considerably high catalytic efficiency albeit its active site is present deep in narrow gorge [19]. Substrate molecule is assisted by extraordinarily high electric field to approach active target site [20,21].

AChE is an efficient serine hydrolase enzyme that degrades acetylcholine neurotransmitter by hydrolysis resulting in termination of impulse signaling at cholinergic synapses [22]. AChE is a major component of various conducting tissues like central and peripheral, nerves and muscles, cholinergic and non-cholinergic fibers and motor and sensory fibers. However the expression of AChE in motor neurons is higher than sensory neurons [23]. Protein structure of AChE (ID: 4BDT) was taken from RCSB PDB. The protein structure was prepared which include protonation via Protonate3D [24] algorithm and AMBER99 force-field was applied for energy minimization.

### Preparation of ligand library

*Beta vulgaris* L. is well known for its health benefits including its antioxidant, antitumor, hepatoprotective, anti-inflammatory and nephroprotective activities. Different plant parts like leaves and roots are widely consumed as vegetables considering its high nutritional value. Various phytochemicals have been extracted and purified from different parts of plant [25].

Different phytochemicals of *B*. *vulgaris* were selected and their structures were extracted through PubChem (https://pubchem.ncbi.nlm.nih.gov/). Water-soluble vitamins like ascorbic acid, niacin, folic acid; phenolic compounds like p-coumaric acid, gallic acid and ferulic acid; flavonoids like myricetin, naringenin, apigenin; betanin (glycosidic food dye) and betaine were selected for anticholinesterase activity (Table 1). Their structures were drawn using Chemdraw software (Chemdraw Ultra 12.0).

**Table 1:** List of phytochemicals selected from B. vulgaris for docking against AChE.

***Water soluble vitamins contents of Beta vulgaris roots***				
**S #**	**Name of compounds**	**Abbreviations**	**IUPAC Names**	
1.	Ascorbic Acid (Vitamin C)	AA	(5*R*)-[(1*S*)-1,2-Dihydroxyethyl]-3,4-dihydroxyfuran-2(5*H*)-one	
2.	Niacin (Vitamin B3)	NI	Pyridine-3-carboxylic acid	
3.	Pyridoxine (Vitamin B6)	PY	(5-hydroxy-6-methylpyridine-3,4-diyl)dimethanol	
4.	Folic acid	FA	(2S)-2-[[4-[(2-Amino-4-oxo-1H-pteridin-6-yl)methylamino]benzoyl]amino]pentanedioic acid	
***Phenolic compounds of Beta vulgaris roots***				
5.	Gallic Acid	GA	3,4,5-Trihydroxybenzoic acid	
6.	Catechol	CL	Benzene-1,2-diol	
7.	p-Coumaric acid	PC	(2E)-3-(4-Hydroxyphenyl)prop-2-enoic acid	
8.	Ferulic acid	FR	(E)-3-(4-hydroxy-3-methoxy-phenyl)prop-2-enoic acid	
9.	o-Coumaric acid	OC	(E)-3-(2-Hydroxyphenyl)prop-2-enoic acid	
10.	Cinnamic acid	CA	(2E)-3-Phenylprop-2-enoic acid	
***Flavonoid compounds of Beta vulgaris root***				
11.	Myricetin	MC	3,5,7-Trihydroxy-2-(3,4,5-trihydroxyphenyl)-4-chromenone	
12.	Naringenin	NA	5,7-Dihydroxy-2-(4-hydroxyphenyl)chroman-4-one	
13.	Kaempferol	KM	3,5,7-Trihydroxy-2-(4-hydroxyphenyl)-4H-chromen-4-one	
14.	Apigenin	AG	5,7-Dihydroxy-2-(4-hydroxyphenyl)-4H-1-benzopyran-4-one	
15.	Betanin(Red glycosidic food dye)	BE	4-(2-(2-carboxy-5-(beta-D-glucopyranosyloxy)-2,3-dihydro-6- hydroxy-1H-indol-1-yl)ethenyl)-2,3-dihydro-(S-(R*,R*))-2,6-pyridinedicarboxylic acid	
16.	Betaine (trimethyl glycine) byproduct of sugar beet processing	BN	2-(trimethylazaniumyl)acetate	
17.	Donepezil (Control)	DP	(RS)-2-[(1-Benzyl-4-piperidyl)methyl]-5,6-dimethoxy-2,3-dihydroinden-1-one	

### Molecular docking

The best poses of docked molecule were generated using Triangular matcher algorithm [26], and grading of simulated poses was achieved through the MOE London dG scoring function through MOE software. For each molecule top 10 ranked poses were generated which were further minimized by Force field refinement algorithm. Moreover, the Generalized Born solvation model was employed for calculation of final binding energy while retaining rigidity of receptor residues. Phytochemicals were categorized based on binding affinity, S-score and Root-Mean-Square Deviation (RMSD) values. The MOE LigX tool was employed for analysis of 2D plots of receptor ligand interactions that enables vivid visualization of docked complexes.

### Molecular dynamics simulations

Molecular dynamics simulation determines dynamic properties of ligand-protein complex regarding free-energy landscape just about the native state of receptor protein within body. Accordingly, MD simulation of complex provides a substantial profile regarding interaction. Betanin, myricetin, betaine and donepezil were subjected to MD simulation using Module Desmond having inbuilt optimized capability for liquid simulation (OPLS 2005) force field at 20 ns. The macromodel Protein Preparation Wizard was used to minimize the protein complex ti ensure the complex arrayed to Desmond. Further, RMSD, ligand root mean square fluctuation (RMSF) and ligand contacts were obtained to make sure the stability of all docked complex in its dynamic conformation along trajectory.

### *In silico* evaluation of drug likeness and ADME/T properties

On the basis of docking score, phytochemicals were further subjected to analyze their pattern to follow Lipinski’s rule of five (Ro5) [27], and compounds with any Ro5 violations were excluded. This was basically done by Molinspiration server [28] for calculation of their physicochemical properties. In order to evaluate drug like characteristics, the candidates were subjected to Swiss ADME software [29]. The calculation of ADMET properties i.e. Absorption, Distribution, Metabolism, Excretion, and Toxicity are an important indication for determining the fate, behavior and toxicity level of a drug candidate in human body. It depicts the feasibility of a drug candidate to pass through the blood-brain barrier, metabolism, its absorption in intestines, distribution at subcellular level and essentially the level of harm it may cause in the body [30].

### *In vitro* acetylcholinesterase assay

Acetylcholinesterase inhibitory activity was determined using Ellman’s spectrophotometric assay [31] with some modifications using acetylcholine chloride as substrate for AChE. The reaction mixture was containing 60 μL phosphate buffer saline (PBS), 10 μL AChE enzyme (0.015 U/well, E.C.3.1.1.7 from electric eel) and phytochemicals (10 μL). After mixing contents, mixture-containing plate was incubated at 37°C for 10 min and absorbance was noted at 405 nm. Further, 10 μL of acetylcholine chloride (0.5 mM) was added followed by the addition of DTNB. Plate was incubated at 37°C for 20 min and absorbance was recorded at 405 nm in a microplate reader. Experiments were performed in triplicate with their respective controls. Donepezil (0.1 mM/well, a reference standard drug) was used as positive control. Percent acetylcholinesterase inhibition was calculated using following formula:

$$Percent\;inhibition=1-\left(\frac{At}{Ac}\right)\times 100$$

Where ‘At’ and ‘Ac’ are the absorbance obtained with and without inhibitors subsequently subtracting the corresponding background.

### Dose response analysis

Dose response assay for AChE inhibition was performed using serially diluted concentrations (400 μM to 12.5 μM) of betanin and betaine. Both compounds were analyzed for their IC_50_ through nonlinear regression analysis.

### Statistical analysis

For analysis of *in vitro* experimental assays, GraphPad Prism 7 software was used. For comparison of treated and non-treated groups, one way ANOVA was performed. For determining the IC_50_ values of compounds, nonlinear regression method was used.

## Results

### Molecular docking and pharmacophore studies

AChE, a serine hydrolase enzyme exhibits structural weight of 72.7 KDa, atomic count 5057, residual count 624 and two distinctive proteins. Enzyme structure was refined after removal of nonstandard residual components. For analysis of enzyme ligand interactions and structure of targeted enzyme with ligand, pre-docking file was submitted to MOE software.

**Fig 1:**
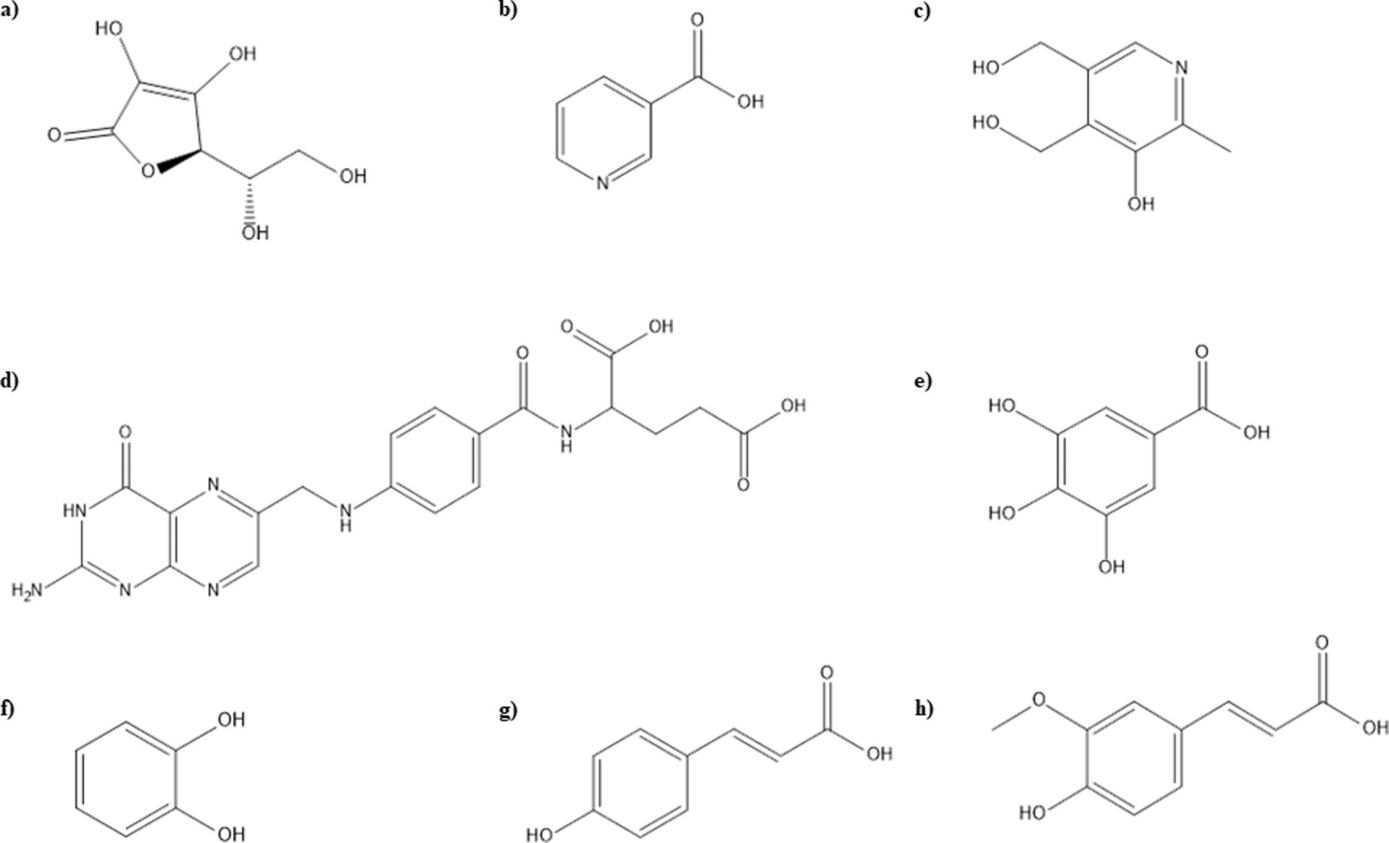
Structures of phytochemicals of *B*. *vulgaris* a) Ascorbic acid **b)** Niacin **c)** Pyridoxine **d)** Folic acid **e)** Gallic acid **f)** Catechol **g)** p-coumaric acid **h)** Ferulic acid.

**Fig 2:**
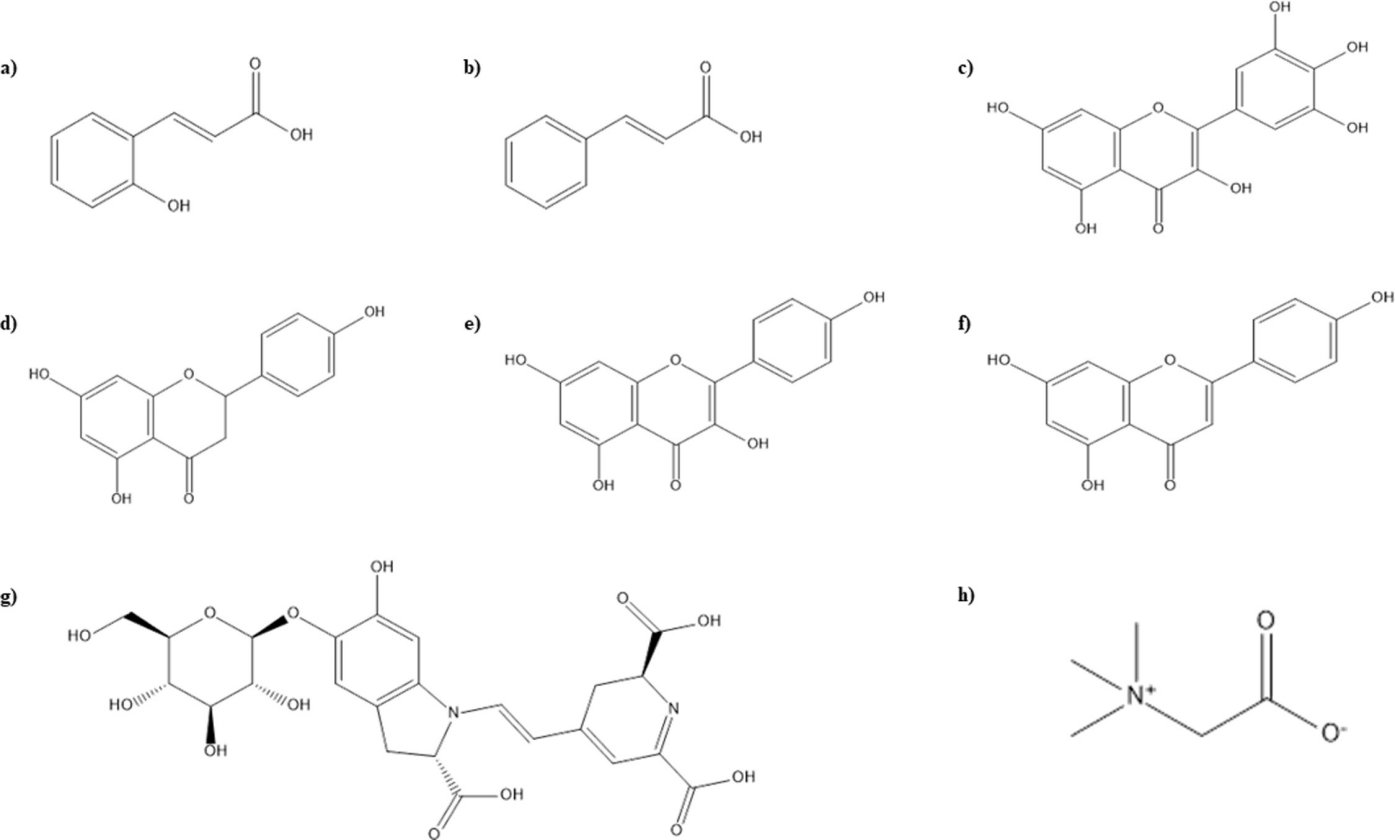
Structures of phytochemicals of *B*. *vulgaris* **a)** o-coumaric acid **b)** Cinnamic acid **c)** Myricetin **d)** Naringenin **e)** Kaempferol **f)** Apigenin **g)** Betanin **h)** Glycine betaine.

Our predictive model is relied on ligand-based pharmacophoric characteristics of phytochemicals. Structures and IUPAC names of beneficial secondary metabolites i.e. betanin, glycine betaine, water soluble vitamins, phenolic and flavonoid compounds present in *B*. *vulgaris* L. (Figs 1 and 2), were extracted through ChemDraw Ultra 12.0 and analyzed using PubChem (Table 1).

Phytochemicals were screened for docking against AChE enzyme and docked complexes were graded based on the stringent filter including four factors like maximum hydrogen bonding interaction, maximum accommodation of binding pocket with minimum free energy and other non-covalent strong interactions. Out of sixteen phytochemicals, all compounds except one followed the Lipinski rule of 5 and preferred for generation of pharmacophoric model (Table 2).

**Table 2:** Results of phytochemicals examined for Lipinski rule.

S #	Compounds abbreviations with PubChem CID	Molecular weight (g/mol)	Number of HBA nOHNH	Number of HBD	MLogP
	Lipinski rule of five	<500	<10	<5	<5
1	AA (54670067)	176.12	6	4	-1.40
2	NI (938)	122.10	3	0	-2.80
3	PY (1054)	169.18	4	3	-0.55
4	FA (135398658)	439.39	13	5	-3.48
5	GA (370)	169.11	5	3	-2.82
6	CL (289)	110.11	2	2	0.99
7	PC (637542)	163.15	3	1	-1.28
8	FR (445858)	193.18	4	1	-1.47
9	OC (637540)	163.15	3	1	-1.04
10	CA (444539)	147.15	2	0	-0.81
11	MC (5281672)	318.24	8	6	1.39
12	AG (5280443)	270.24	5	3	2.46
13	BE (6540685)	545.43	15	3	-4.64
14	BN (247)	117.15	3	0	-5.41
15	DP (3152)	380.51	4	1	1.14

**Fig 3:**
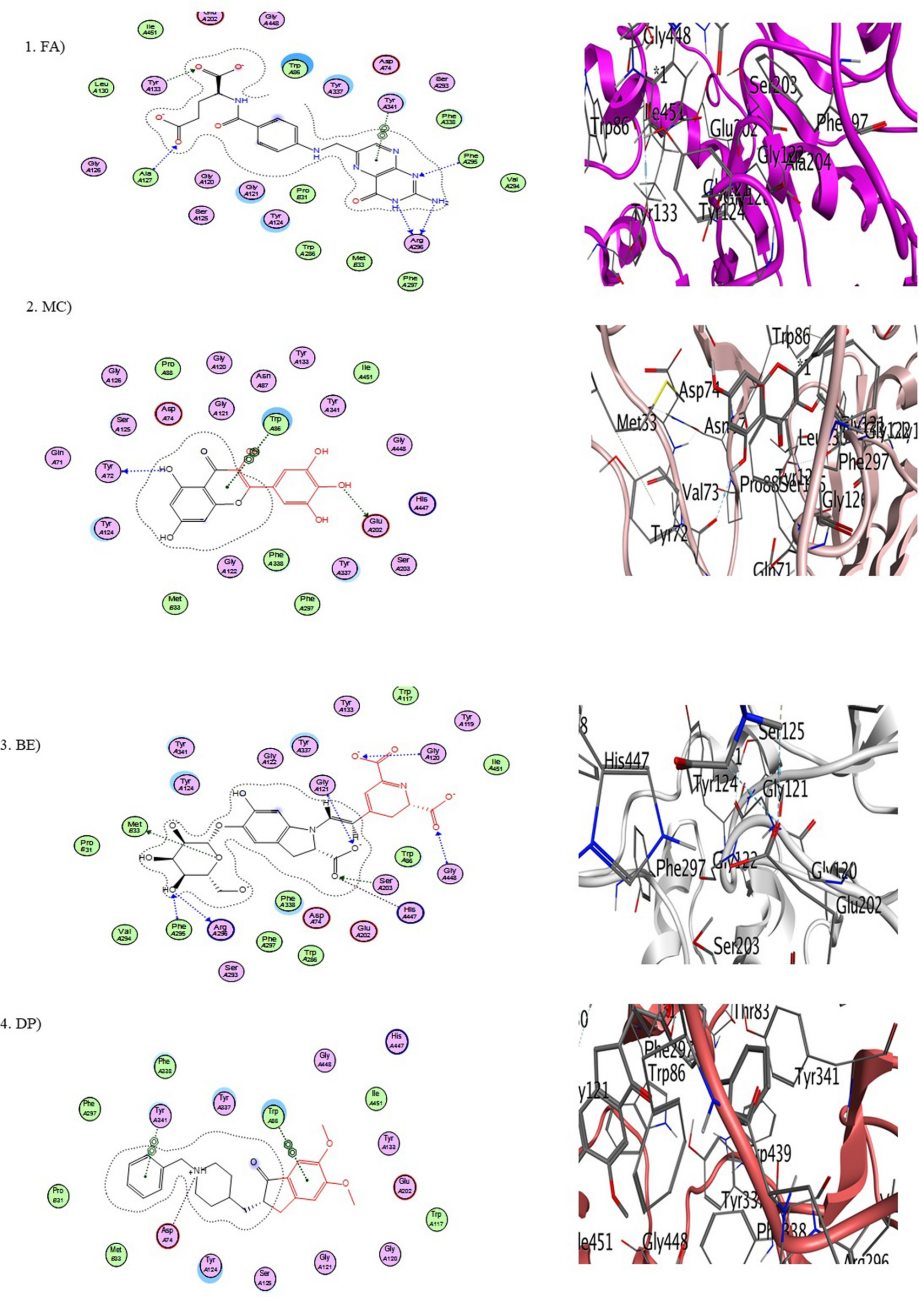
a) Enzyme ligand interactions within the binding domain of AChE for folic acid (FA) and myricetin (MC) b) Enzyme ligand interactions within the active binding domain of AChE for betanin (BE) and donepezil (DP).

Determining the details of ligand-protein binding interactions may help predict promising bioactivity at the early stage of the drug discovery process. To validate our predictive model, molecular docking was performed through MOE software to intend the significantly strong binding interactions. Docking pattern between enzyme and each phytochemical was analyzed to determine the ligand binding sites. After analysis, three ligands were successfully bound to active binding domains of targeted enzyme as illustrated in Fig 3A and 3B. Docking study depicts all interacting residues of enzymes and strong van der Waals forces. Sixteen selected phytochemicals of *B*. *vulgaris* L. along with donepezil (standard drug) were docked against AChE enzyme. Out of sixteen, three compounds, betanin, myricetin and folic acid exhibited minimum binding energy in the range of -22 kcal/mol to -16 kcal/mol in comparison to reference drug donepezil (-17 kcal/mol). Least binding energy and scoring function of every docked ligand is mentioned in Table 3.

**Table 3:** Interaction details of phytochemicals in the proposed site of AChE enzyme.

S #	PubChem Id	Compounds name	Docking score (kcal/mol)	Interaction detail		
				RMSD value	Residues	Interaction
1	54670067	Ascorbic acid	-10	0.7	Glu202	H-acceptor
2	938	Niacin	-7	1.8	Ser203 Gly121	H-donor H-acceptor
3	1054	Pyridoxine	-10	2.2	Glu202 Gly121	H-acceptor H-donor
4	135398658	Folic acid	-19	1.1	Arg *A*296 Tyr *A*133 Ala *A*127 Tyr *A*341	H-acceptor H-donor H-donor pi-pi
5	370	Gallic acid	-12	-0.8	Glu202 Gly121	H-acceptor pi-H
6	289	Catechol	-7.0	1.2	Glu202 Gly121	H-acceptor pi-H
7	637542	p-Coumaric acid	-10	0.9	Ser203 His447	H-donor H-donor
8	445858	Ferulic acid	-10	1.3	Gly121 Glu202 Tyr124	pi-H H-acceptor H-donor
9	637540	o-Coumaric acid	-9.0	1.3	Tyr *A*341	pi-pi
10	444539	Cinnamic acid	-8.0	1.7	Gly121 Tyr124	pi-H H-donor
11	5281672	Myricetin	-16	1.3	Trp*A*86 Glu*A*202	pi-pi H-acceptor
12	932	Naringenin	-13	1.6	Phe*A*338	pi-pi
13	5280863	Kaempferol	-14	1.2	Gly121 Glu202 Tyr124	pi-H H-acceptor H-acceptor
14	5280443	Apigenin	-13	1.6	Trp86 Tyr345 Glu202	pi-pi pi-H H-acceptor
15	6540685	Betanin	-22	1.6	Gly120 Gly*A*448 GlyA121 Ser*A*203 His*A*447 Met*B*33	H-donor H-donor H-donor H-donor H-donor H-acceptor
16	247	Betaine	-8.0	1.6	Glu202	H-acceptor
17	3152	Donepezil	-17	1.6	Trp*A*86 Tyr*A*341 Asp*A*74	pi-pi pi-pi H-acceptor

The 2.0D diagrams of protein ligand interactions revealed all interacting binding agents of enzyme and effective van der Waal forces also illustrated in Fig 3A and 3B. Docking results of three selected phytochemicals of *B*. *vulgaris* L. with binding pocket of AChE targeted enzyme (Fig 3A and 3B) illustrate that; (2S)-2-[[4-[(2-Amino-4-oxo-1H-pteridin-6-yl) methylamino] benzoyl] amino] pentanedioic acid (folic acid) interacted with Arg A: 296, Tyr A: 133, Ala A: 127, Tyr A: 341 (Fig 3A), while 3,5,7-Trihydroxy-2-(3,4,5-trihydroxyphenyl)-4-chromenone (myricetin) interacted with Trp A: 86 and Glu A: 202 (Fig 3A). Likewise 4-(2-(2-carboxy-5-(beta-D-glucopyranosyloxy)-2,3-dihydro-6- hydroxy-1H-indol-1-yl)ethenyl)-2,3-dihydro-(S-(R*,R*))-2,6-pyridinedicarboxylic acid (Betanin) showed strong interaction with Gly120, Gly A: 448, Gly A: 121, Ser A: 203, His A: 447, Met B: 33 (Fig 3B) and standard drug, donepezil had interaction with Trp A: 86, Tyr A: 341 and Asp A: 74 (Fig 3B). Docking results depicted the strong binding affinities of ligand molecules with targeted AChE binding domains.

### *In vitro* AChE inhibitory activities of phytochemicals

**Fig 4:**
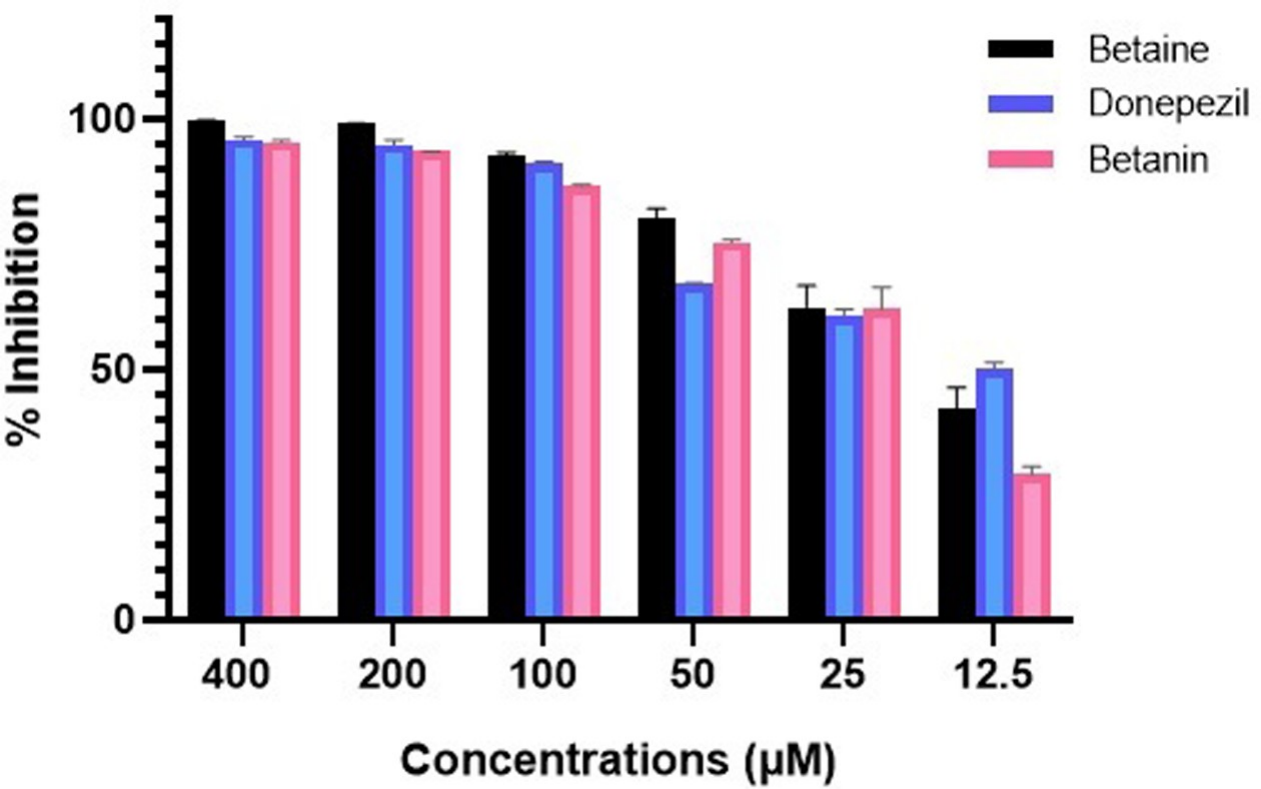
Percentage inhibition of AChE activity from 12.5–400 μM concentration of betanin, glycine betaine and the reference standard drug, donepezil. Results are presented as mean ± SEM for experimental triplicates ****P < 0.0001; **P = 0.0014.

**Fig 5:**
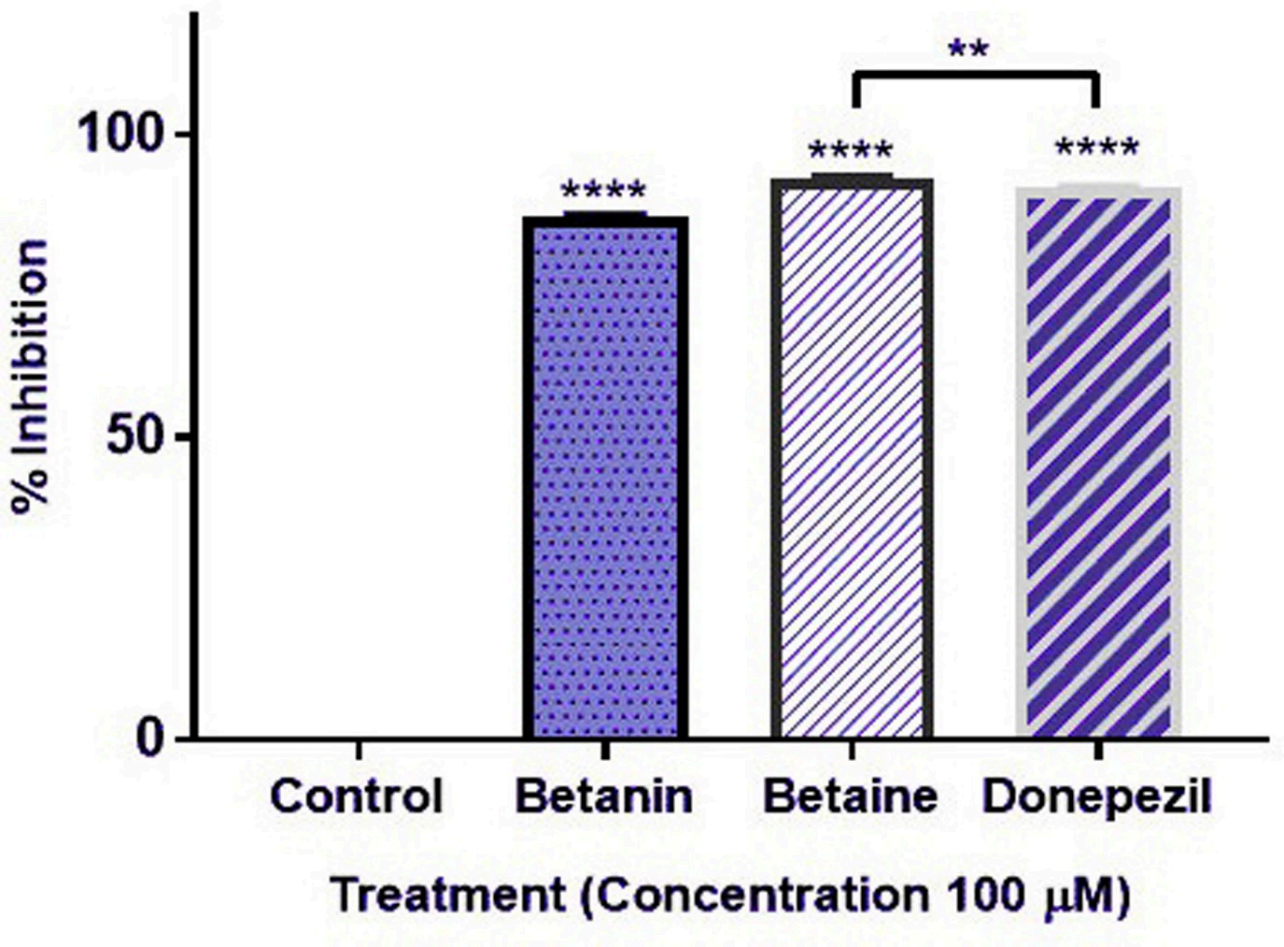
Percentage inhibition of AChE activity at 100 μM concentration of betanin, glycine betaine and the reference standard drug, donepezil. Results are presented as mean ± SEM for experimental triplicates.

**Fig 6:**
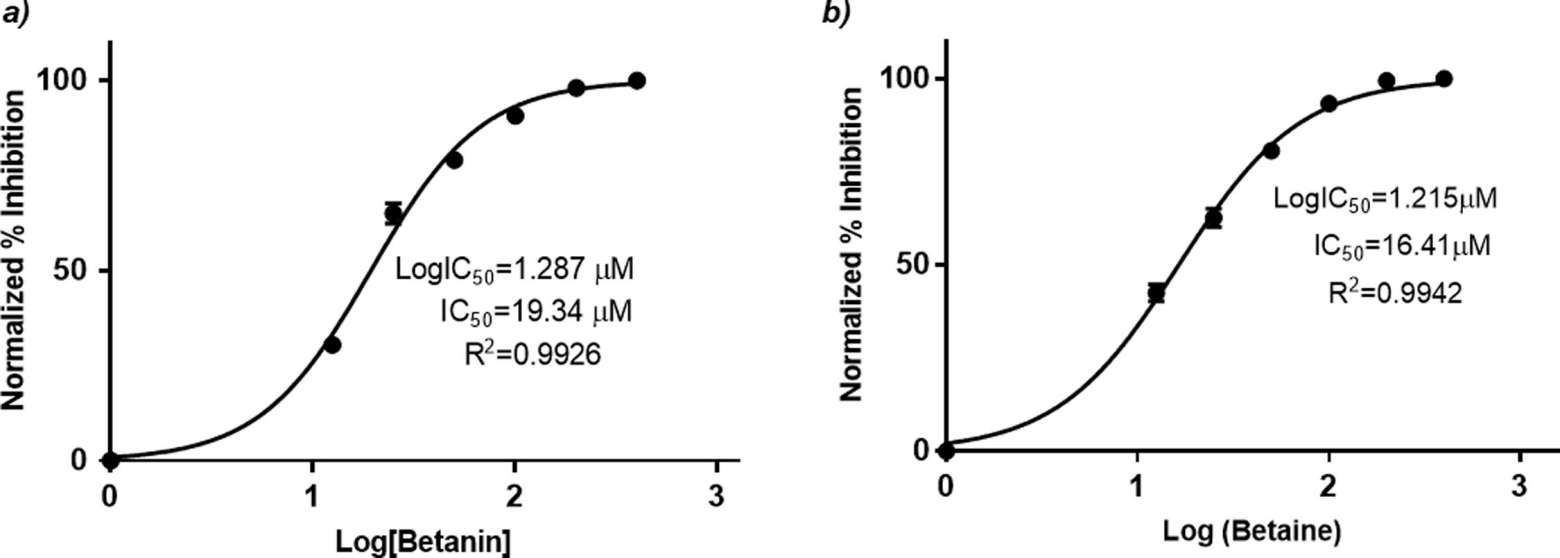
a) Dose response analysis of betanin was performed using serial dilutions (12.5 μM– 400 μM). Nonlinear regression analysis depicted 19.34 μM concentration as IC_50_. b) Dose response analysis of glycine betaine was performed using serial dilutions (12.5 μM– 400 μM). Nonlinear regression analysis depicted 16.41 μM concentration as IC_50_.

**Fig 7:**
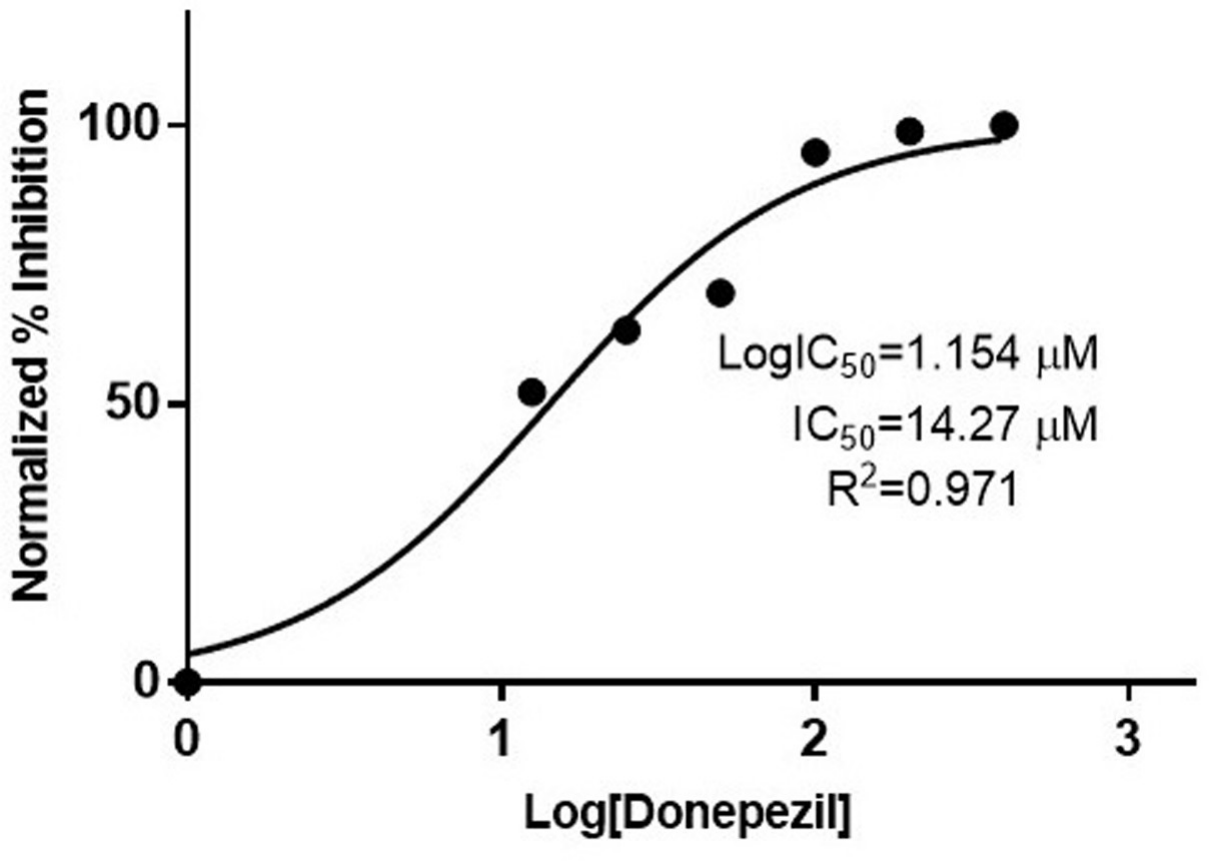
Dose response analysis of standard reference donepezil was performed using serial dilutions (12.5 μM– 400 μM). Nonlinear regression analysis depicted 14.27 μM concentration as IC_50_.

Further, *in vitro* studies were documented for analysis of anti-AChE activity of phytochemicals of *B*. *vulgaris* L. For this purpose, Ellman’s spectrophotometric assay was performed (Fig 4). At concentration of 100 μM, glycine betaine and betanin possessed 92.9% and 86.6% AChE inhibition as compared to donepezil (91.1%) (Fig 5). Dose dependent assay illustrated that glycine betaine and betanin exhibited strong potential against AChE with IC_50_ values of 16.41 μM and 19.34 μM respectively in comparison to donepezil (IC_50_: 14.27 μM). Non-linear regression analysis depicted the LogIC_50_ values for glycine betaine (1.215 ± 0.0147 μM), betanin (1.287 ± 0.0143 μM) (Fig 6A and 6B) and donepezil (1.154 ± 0.040 μM) (Fig 7). Results for AChE inhibition illustrated the significant bioactivity of betanin (*P* < 0.0001) as compared to donepezil (Figs 4 and 5).

### Molecular dynamics simulation

#### Root Mean square deviation (RMSD)

**Fig 8:**
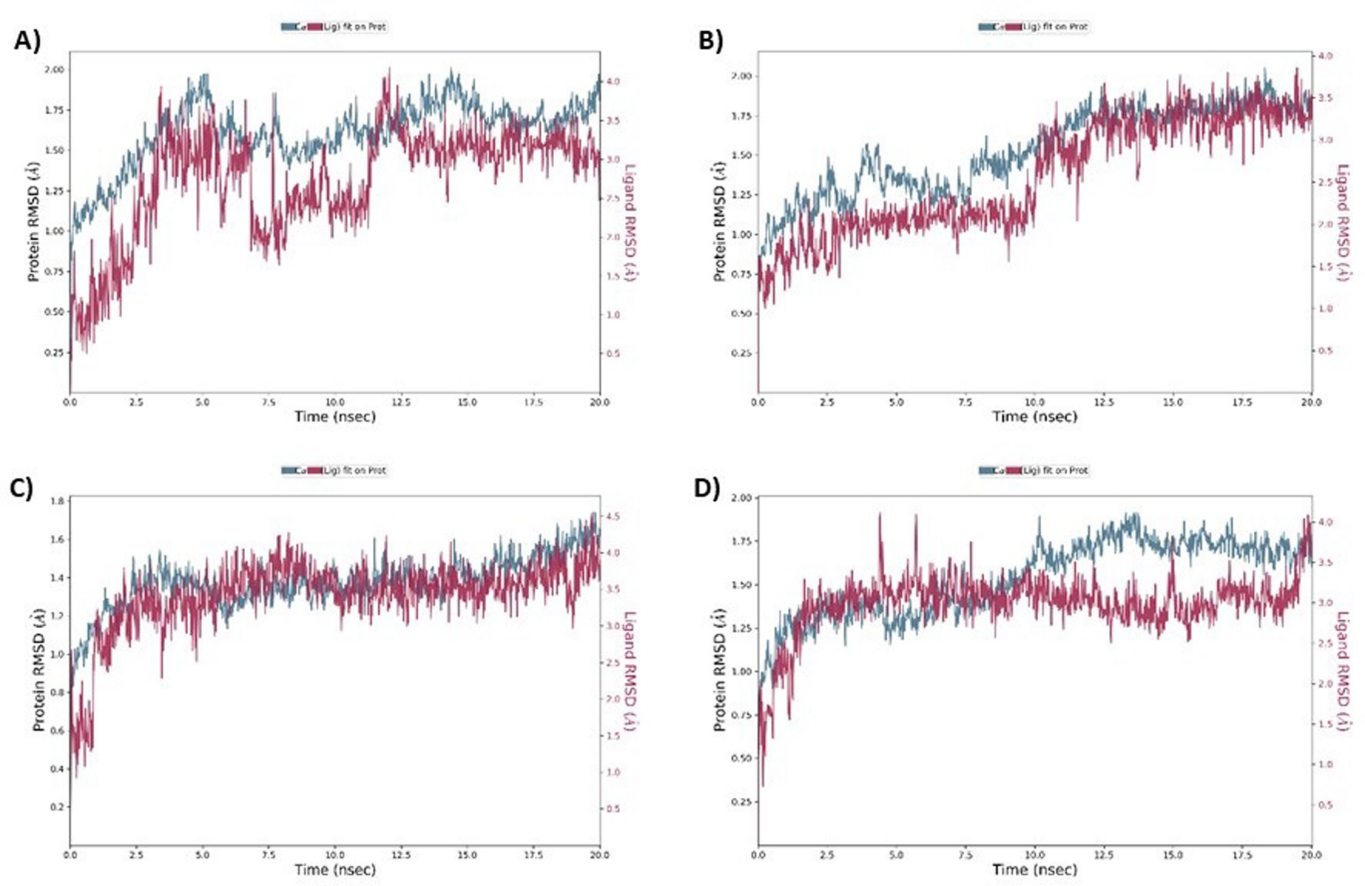
MD simulation interaction diagrams of 20 ns trajectory showing RMSD plot for complex acetyl cholinesterase- Betanin (A), acetyl cholinesterase- Betaine (B), acetyl cholinesterase- Myricetin (C) and acetyl cholinesterase-Donepezil (D) respectively.

To evaluate conformational dynamics of protein-ligand complexes up to 20 ns, MD simulation was done to find RMSD values RMSD plots for complex of acetyl cholinesterase with Betanin showed the fluctuation of 2.5 nsec, 5 nsec and 12 nsec and the stable trajectory throughout the production run with maximum deviation of 1.9 Å. RMSD value was between 0.50 to 1.75 Å for the complex (Fig 8A). The RMSD plot for the complex of acetyl cholinesterase with Betaine showed the fluctuation maxima at up to 10ns and acquired stability beyond 10 ns simulation interval and RMSD value was between 0.60 to 1.9 Å for both protein and ligand, indicating a stable complex between them (Fig 8B). Moreover, RMSD for acetyl cholinesterase complex with Myricetin (Fig 8C) depicted intense deviation at 1.5ns followed by stabilization throughout the interval. The complex acetyl cholinesterase complex with Donepezil showed fluctuation at 5 nsec and 6.5 nsec (Fig 8D) and depicted stability beyond this.

#### Root-mean-square fluctuation (RMSF)

**Fig 9:**
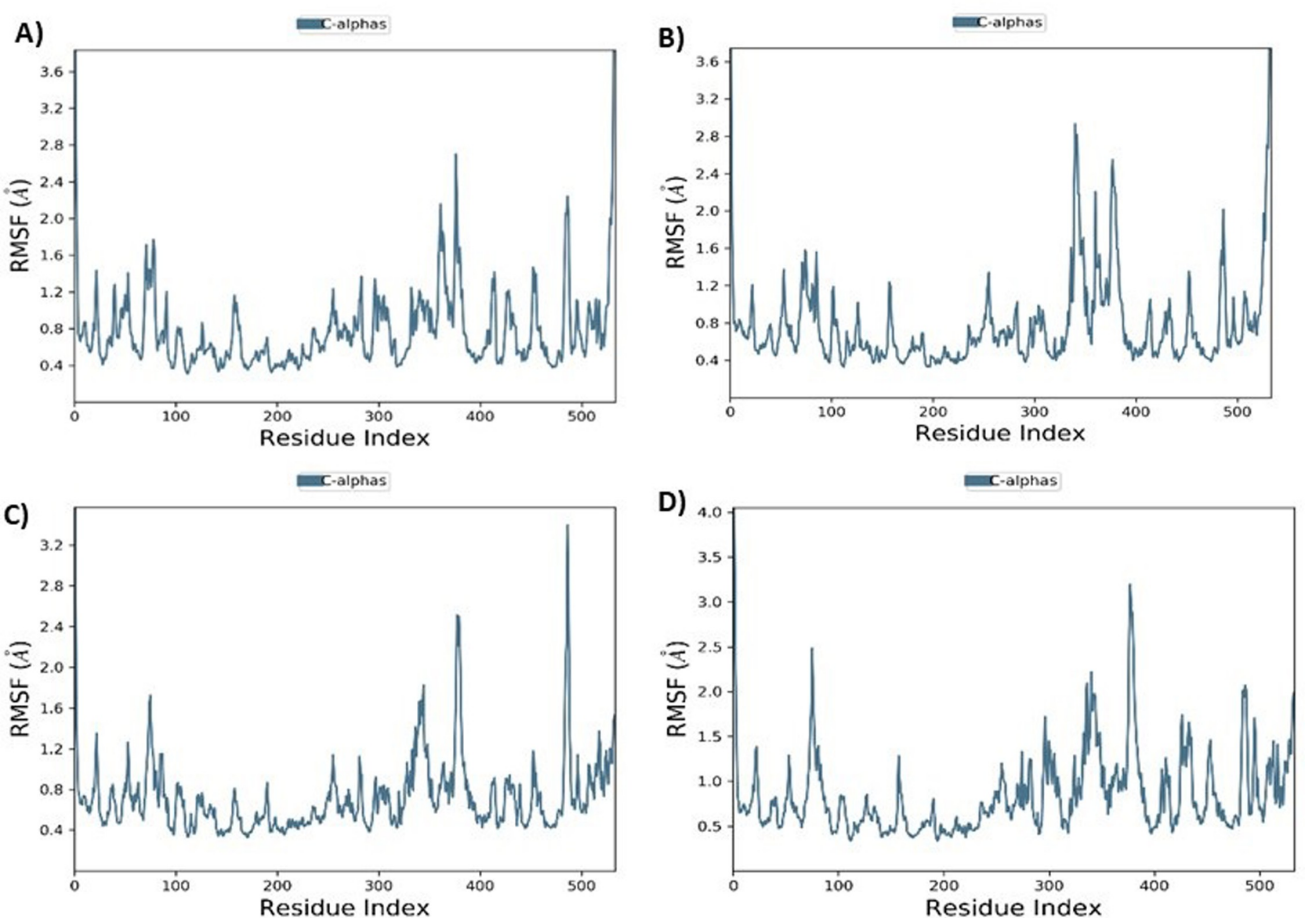
Protein Root Mean Square Fluctuation (RMSF) plots (Angstrom). (A) RMSF trajectory plot of acetyl cholinesterase- Betanine complex showing residue-wise fluctuation, (B) RMSF trajectory plot for acetyl cholinesterase- Betaine complex showing residue-wise fluctuation, (C) RMSF trajectory plot of acetyl cholinesterase with Myricetin complex showing residue-wise fluctuation, (D) RMSF trajectory plot of acetyl cholinesterase with Donepezil complex showing residue-wise fluctuation.

The RMSF peaks calculate the area of protein where residues fluctuate maximum over the simulation trajectory. The RMSF peaks of complexes are shown in Fig 9. The RMSF of individual amino acid residues of the protein were computed during the entire simulation process to ascertain the flexibility of protein system. The RMSF of acetyl cholinesterase complexes ranged from 0.5 to 2.5 Å and 0.5 to 4.5 Å with the local ligand-contact maxima at 2.7 Å and 2.9 Å for Betanine and Betaine respectively Fig 9A and 9B. Similarly, the RMSF of acetyl cholinesterase complexes ranged from 0.6 Å to 3.4 Å and 0.4 to 3.1 Å with the local ligand-contact maxima at 0.8 Å and 1.5 Å for Myricetin and Donepezil respectively Fig 9C and 9D. Additionally, atoms in also depicted the acceptable and stable RMSF fluctuations during the simulation interval. These observations implies that these proteins have attained a relatively stable complex system with the respective ligands.

#### Protein-ligand interaction

**Fig 10:**
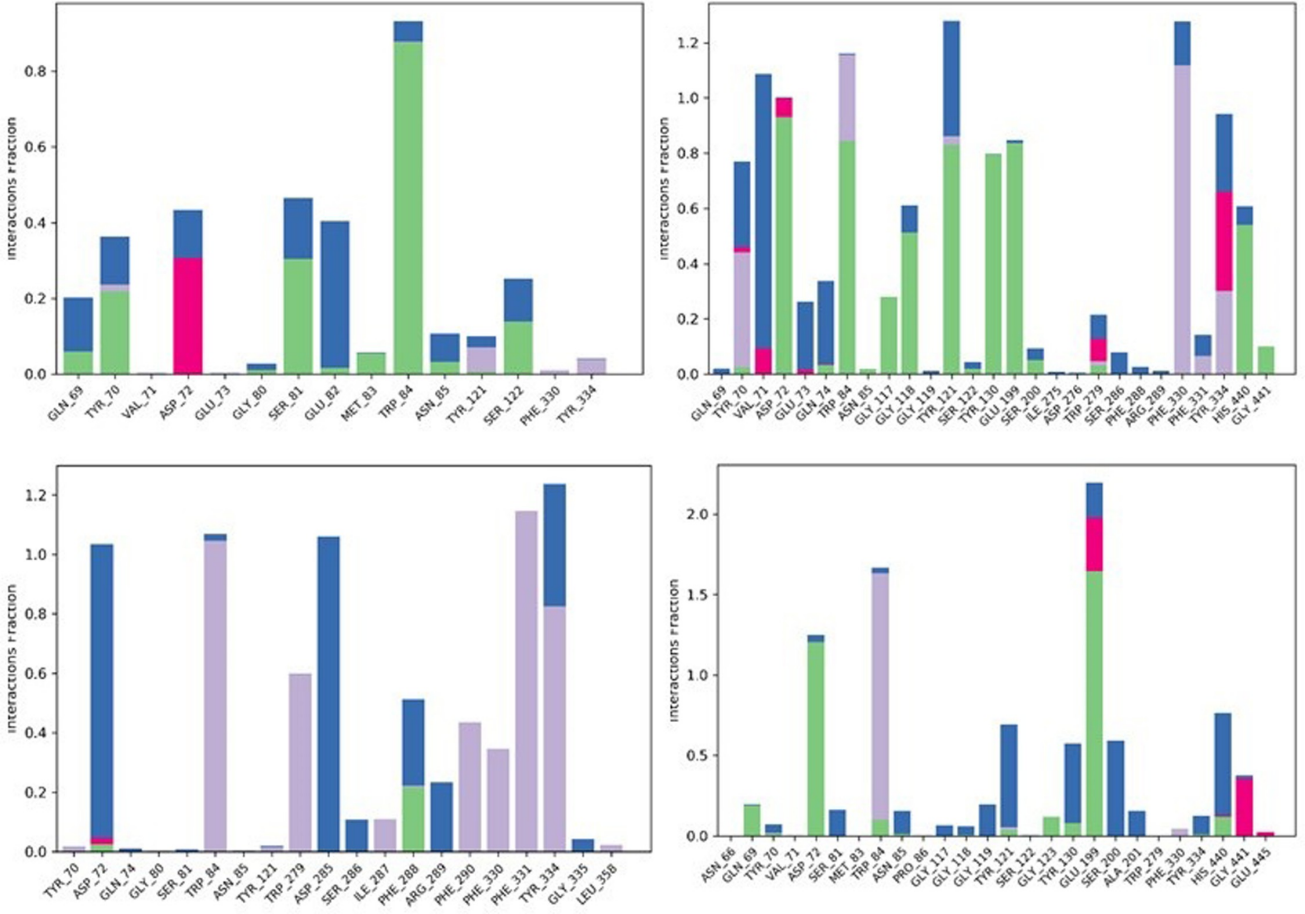
Histogram (stacked bar chart) showing forming H-bonds interactions (green color), hydrophobic interactions (gray violet color), and water bridges (blue color) during 50 ns simulation for complex acetyl cholinesterase-Betanin (A), acetyl cholinesterase- Betaine (B), acetyl cholinesterase- Myricetin (C) and acetyl cholinesterase-Donepezil (D).

The various intermolecular interactions, such as H-bonds, H_2_O bridges, hydrophobic and ionic interactions, were calculated over 20ns of the MD simulation analysis The data showed that acetyl cholinesterase made strong H-bonding with amino acids TRP 84, SER181 and SER122. It also showed strong hydrophobic interactions with ASN 85 and TYR334 and water bridge with GLU82 as depicted in Fig 10A. It has been observed that the two residues PHE 330 and TYR 70 exhibited hydrophobic interaction for the acetyl cholinesterase- Betaine complex, and ionic bond as well as water bridge with residue VAL71.The amino acids including TYR 130 and GLU 199 participated in hydrogen bonding as shown in Fig 10B. The acetyl cholinesterase- Myricetin interaction map predicted during the simulation showed participation of PHE 288 in hydrogen bonding, PHE290, PHE 330, PHE 331 and TRP 279 in hydrophobic and ASP 285 in water bridging as shown in Fig 10C. The Myricetin also showed strong hydrophobic interactions with TRP 84 and ionic strength with GLY441. This potent compound also showed water bridge interaction with SER 200 and ALA 201 (Fig 10C).

#### Radius of gyration (Rg) and solvent accessible surface area (SASA)

**Fig 11:**
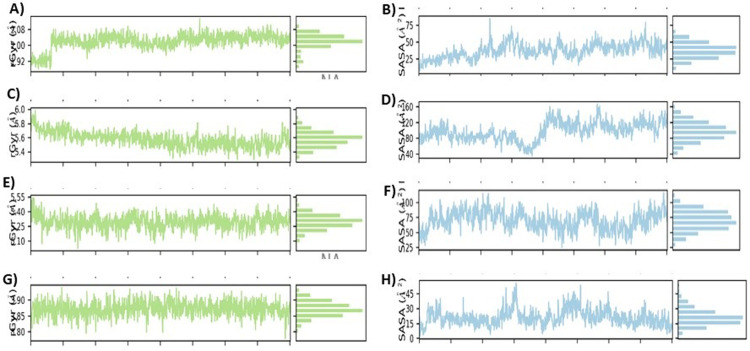
The time frame evolution against the radius of gyration (Rg) (A, C, E and G) displayed on left and the SASA plots of docked complexes over 50 ns MD simulation (B, D, F and H) displayed on right.

The radius of gyration (Rg) is a parameter for analyzing the equilibrium confirmation of protein structure during simulation. The Fig 11A, 11C, 11E and 11G display Rg values of the acetyl cholinesterase- Betanin complex, acetyl cholinesterase- Betaine complex, acetyl cholinesterase- Myricetin complex and acetyl cholinesterase-Donepezil complex during the MD trajectory pose with corresponding Rg values through the simulation at 20 ns were 0.16 ± 0.34 nm, 0.23 nm ± 0.26 nm, 0.23 ± 0.24 nm and 0.32 nm ± 0.34 nm, respectively.

The Solvent accessible surface area (SASA) analysis measures the interaction between complexes and solvents. The estimated average range SASA values of the acetylcholinesterase- Betanin complex, acetylcholinesterase- Betaine complex, acetylcholinesterase- Myricetin complex, acetyl cholinesterase-Donepezil complex for 20 ns simulation were between the 0.3 ± 0.9 nm, 0.1 ± 0.4, 0.1 ± 0.4 nm and 0.1 ± 0.5 nm respectively as depicted in Fig 11B, 11D, 11F and 11H. The results suggested that it should be accessible for solvents and have more interaction with solvents. In addition, SASA values for the three protein complexes remained stable during MD simulation run.

## Discussion

Plants utilization in traditional medicine for treating multiple ailments is an indispensable element of culture and tradition of majority of world’s population. Additionally, multiple aspects like availability, accessibility and affordability of traditional medicinal plants make high demand of this therapeutic approach [32]. Secondary metabolites like phenolic compounds, flavonoids, alkaloids, saponins, tannins etc. are produced by plants for their defensive mechanism and implicated as therapeutic agents [33]. *B*. *vulgaris* L. is a vegetable and its different parts have long been utilized as traditional medicine for the cure of multiple ailments. AD is one of the major medical care challenges in therapeutic research and is the foremost reason of causing dementia. Ailment-modifying treatment approaches for AD are albeit under broad-spectrum research studies. Currently, AD treatments include symptomatic treatments exclusively and assist in compensating the clinical symptoms. In continuing clinical trials, researchers are testing multiple possible therapeutic agents targeting multiple factors like neurotransmitter modifications, neuro-inflammation, amyloid and tau aggregation [34]. Targeting AChE inhibition is considered to be a competent therapeutic approach in AD management according to cholinergic hypothesis [35]. Due to inhibition of AChE, the levels of acetylcholine, a neurotransmitter, increases in the brain thereby improving cholinergic functions in AD patients and mitigate the symptoms of neurological disorders [36]. Therefore, variety of plants and their derived compounds with negligible side effects have been used for the management of AD by blocking AChE.

Current research work includes the *in silico* and *in vitro* study for the investigation of anti-acetylcholinesterase effect of phytochemicals of *B*. *vulgaris* L. Structures of phytochemicals were extracted from PubChem and toxicity analysis was performed. Drug-likeness filter depicts the least toxicity of phytochemicals except one. To analyze the drug likeness characteristics of all sixteen phytochemicals, multiple computational filters including MlogP and predicted solubility were utilized to opt the correct phytochemicals. Generally, phytochemicals followed the Lipinski’s rule of 5 were further processed to scrutinize their catalytic potentials. Computational analysis of ADME/T profile for all phytochemicals were performed along with depiction of free binding energies. The targeted characteristics including MlogP, hydrogen bond donor atom (HBDH), molecular weight and hydrogen bond acceptor atom (HBAH) were successfully elucidated (Table 2). Docking studies of phytochemicals with AChE depicts their strong binding within enzyme domain. Docking results for betanin, myricetin and folic acid indicated their minimal binding score (< -18) as compared to standard drug, donepezil (Table 3). Previous *in silico* studies are in line with our study revealed the interaction of drugs with AChE not only elevate the acetylcholine levels but also reduce Aβ accumulation in *Caenorhabditis elegans* [37]. Our study depicts that few of phytochemicals are highly selective to their targeted enzyme. Betanin, myricetin and folic acid significantly docked the AChE enzyme. Though RMSD values for betanin, betaine, myricetin complexes are high, the hydrophobic interactions of multiple residues for all these complexes were reproduced through docking and simulation studies presenting a significant binding affinity towards AChE. The output of parameters measured from MD simulation demonstrated that values were narrowly diversified within an acceptable range during simulation time indicating steadiness in complexes conformation. Previously, several studies are reported for describing potential role of MD simulation in drug designing. In a study, reported by Khalid et al, a series of synthetic compounds were evaluated for their anti-HCV NS5B polymerase activity through molecular docking and simulation studies [38]. The 3.0D structural conformation of pharmacophore model is depicting the key features like HBA, HBD, hydrophobic, aromatic ring, positive ionizable component for ligand binding (Fig 3A and 3B). Serial dilutions (400 μM—12.5 μM) of betaine, betanin and donepezil were analyzed for their anti-AChE activity (Fig 4). At 400 μM and 200 μM dose concentration of glycine betaine, AChE was inhibited maximally to 99% while at the same concentrations, betanin and donepezil depicted 98% anti-AChE activity (Fig 4). Results depicted that both phytochemicals exhibited strong anti-AChE inhibitory activity comparable to standard drug, donepezil (Fig 4). Current research study is strong evidence for the inhibition of AChE ultimately helpful for the cure of Alzheimer’s disease in future after conducting further studies. The *in vitro* studies indicated the significant inhibition of AChE in the presence of betanin and glycine betaine. Results indicated the 19.34 μM and 16.41 μM IC_50_ values for betanin and glycine betaine that are comparable to donepezil IC_50_ value of 14.27 μM (Figs 6 and 7). Nonlinear regression analysis depicted the best-fit values for phytochemicals against AChE (Table 4). IC_50_ of both phytochemicals, glycine betaine and betanin delineated strong therapeutic efficacy via inhibiting AChE, comparable to standard drug, donepezil (Figs 6 and 7). In future, these predicted pharmacophore characteristics along with *in vitro* studies of phytochemicals would assist to identify new drugs against AD. Molecular dynamics simulation studies further supported findings of current research study. Myricetin, betanin, and betaine strongly inhibited AChE activity (Figs 8–11). Solvent accessible surface area (SASA) analysis is used to measure the interaction between solvents and complex. Results suggest that it should be attainable for solvents and exhibit strong interaction with solvents. Additionally, SASA values for the complexes remained stable during the experimental run of MD simulation (Fig 11). Moreover, current *in silico* and *in vitro* study revealed that such research contributions would significantly improve in drug designing and selection of natural/synthetic compounds against various diseases like neurodegenerative disorders, cancer, viral and other microbial infections. Additionally, therapeutic efficacy of natural/synthetic compounds against AD might be assessed by *in vivo* assays.

**Table 4:** IC_50_ values of phytochemicals along with reference standard in AChE inhibitory assays.

Samples	IC_50_ value (μM)	LogIC_50_ (μM)	*R* ^*2*^
Betanin	19.34	1.287	0.9926
Glycine Betaine	16.41	1.215	0.9942
Donepezil	14.27	1.154	0.971

## Conclusion

The *in vitro* and *in silico* pharmacophore, molecular docking and simulation studies of *B*. *vulgaris* phytochemicals have shown potential bioactivity against acetylcholinesterase. Our study scrutinized betanin, myricetin and glycine betaine as potential acetylcholinesterase inhibitors. Our study provided the framework for synthetic modification of phytochemicals, de novo development of structural derivatives and *in vivo* pharmacological activities of betanin, myricetin and glycine betaine. Our study suggests further *in vitro* and *in vivo* experimental pre-clinical trials to analyze the therapeutic efficacy of glycine betaine and betanin in future.

## Supporting information

S1 Graphical abstract (JPG)
