# Supplementary figures and images for: The Insight of In Silico and In Vitro evaluation of Beta vulgaris phytochemicals against Alzheimer’s disease targeting acetylcholinesterase
Source: PLoS One. 2022 Mar 3;17(3):e0264074. doi: 10.1371/journal.pone.0264074 (PMC8893657; doi:10.1371/journal.pone.0264074)

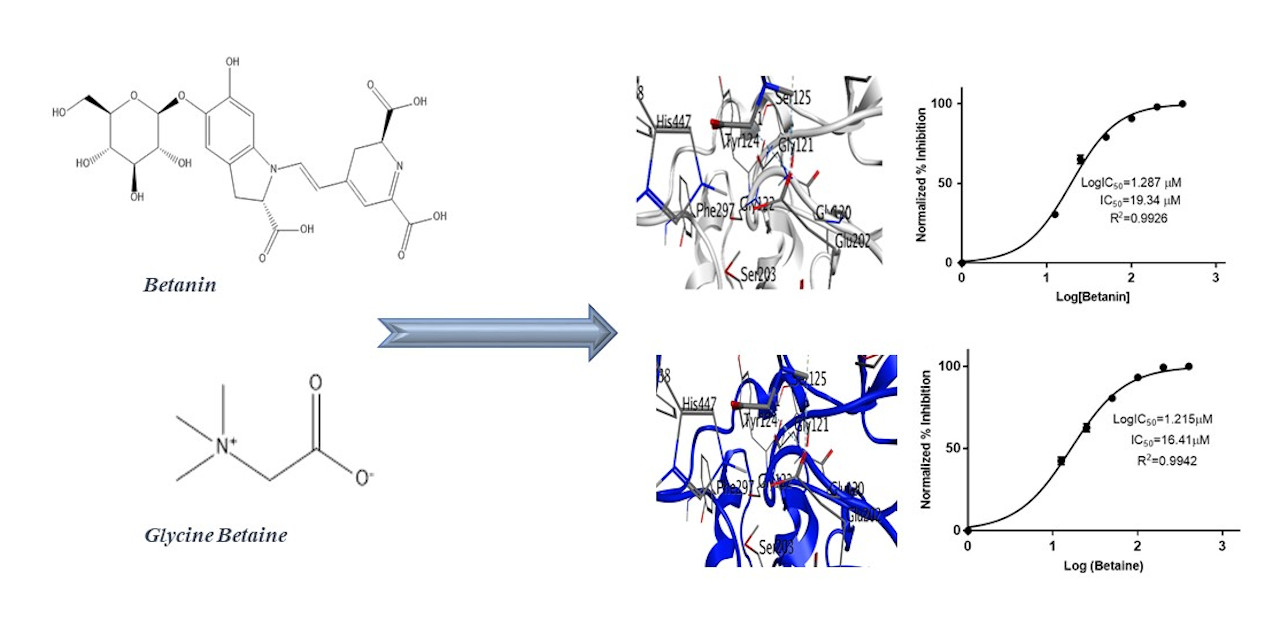

Supplement: S1 Graphical abstract — (JPG) [file pone.0264074.s001.jpg]
